# Influence of Organizational Issues on Nurse Administrators’ Support to Staff Nurses’ Use of Smartphones for Work Purposes in the Philippines: Focus Group Study

**DOI:** 10.2196/17040

**Published:** 2020-01-10

**Authors:** John Robert Bautista, Trisha T C Lin, Yin-Leng Theng

**Affiliations:** 1 School of Information The University of Texas at Austin Austin, TX United States; 2 Center for Health Communication Moody College of Communication The University of Texas at Austin Austin, TX United States; 3 Department of Radio & Television College of Communication National Chengchi University Taipei Taiwan; 4 Taiwan Institute for Governance and Communication Research Taipei Taiwan; 5 Centre for Healthy and Sustainable Cities Wee Kim Wee School of Communication and Information Nanyang Technological University Singapore Singapore

**Keywords:** BYOD, IT consumerization, nurse administrators, organizational support, Philippines, smartphone

## Abstract

**Background:**

Studies show that nurses use their own smartphones for work purposes, and there are several organizational issues related to this. However, it is unclear what these organizational issues are in the Philippines and the influence they have on nurse administrators’ (ie, superiors) support to staff nurses’ (ie, subordinates) use of smartphones for work purposes.

**Objective:**

Drawing from the Organizational Support Theory (OST), this study aimed to identify organizational issues that influence nurse administrators’ support to staff nurses’ use of smartphones for work purposes.

**Methods:**

Between June and July 2017, 9 focus groups with 43 nurse administrators (ie, head nurses, nurse supervisors, and nurse managers) were conducted in 9 tertiary-level general hospitals in Metro Manila, the Philippines. Drawing from OST, issues were classified as those that encouraged or inhibited nurse administrators to support nurses’ use of smartphones for work purposes.

**Results:**

Nurse administrators were encouraged to support nurses’ use of smartphones for work purposes when (1) personal smartphones are superior to workplace technologies, (2) personal smartphones resolve unit phone problems, and (3) policy is unrealistic to implement. Conversely, issues that inhibited nurse administrators to support nurses’ use of smartphones for work purposes include (1) smartphone use for nonwork purposes and (2) misinterpretation by patients.

**Conclusions:**

Nurse administrators in the Philippines faced several organizational issues that encouraged or inhibited support to staff nurses’ use of smartphones for work purposes. Following OST, the extent of their support can influence staff nurses’ perceived organizational support on the use of smartphones for work purposes, Overall, the findings highlight the role and implication of organizational support in the context of smartphone consumerization in hospital settings, especially in developing countries.

## Introduction

### Background

It is now common for employees to use their personal digital devices to accomplish work-related tasks. Harris et al [1] attribute this situation as information technology (IT) consumerization or the “adoption of consumer devices and applications in the workforce.” Among personal digital devices, it is not surprising to see the adoption of smartphone use for work purposes, considering that its portability blurs the boundaries between personal and professional use [2,3]. Instead of using company-issued devices for accomplishing work-related tasks, organizations have observed that employees prefer to use their own devices such as smartphones. For instance, a survey shows that 67% of IT employees are using personal devices in the workplace [4]. Similarly, Intel reported that the number of their employees using their own smartphones for work purposes increased from 3000 in 2010 to 17,000 in 2011 [5]. Although there are organizations that would support their employees’ use of smartphones, there are others that would not support such an initiative [6].

Take the case of health care organizations. A 2015 survey found that 73% of health care organizations across North America allowed their health care staff to bring their own devices (primarily smartphones) for work purposes, but only 51% allowed their nurses to use personal devices for work compared with 91% of medical doctors [7]. Likewise, a recent study in Saudi Arabia showed that although 97% of surveyed health care workers owned a smartphone, only 42% used it for clinical work [8]. It is important to note that about 77% of the respondents in that study were nurses, which suggests the possibility of such restrictions being placed on nurses. Currently, much of the literature focuses on medical doctors’ use of smartphones [9-12]. However, more studies are needed to understand the perspectives of nurses—the largest group of health care professionals in a hospital [13]—as they tend to experience low support on using mobile devices [14,15] despite the potential benefits it could bring to enhancing clinical work and improving patient care [16-22].

Considering that IT consumerization in health care organizations is a pertinent issue faced by several levels of hospital administrators [23], it is crucial to understand specific issues encountered by nurse administrators when their staff nurses use their smartphones for work purposes. To date, studies that present issues related to nurses’ use of smartphones for work purposes (eg, distraction, potential for medical errors, reduced quality of care, privacy and confidentiality issues, and nomophobia) were derived from surveys or interviews with bedside nurses [20,22] or nursing students [15,24-27]. Thus, there is a need for studies that focus on the perspectives of nurse administrators. More importantly, there’s a need for studies that put forth the point of view of nurse administrators because nurse administrators are in the best position to provide insights on how to appropriate implicit or explicit *bring your own device* (BYOD) policies for staff nurses given the manpower and technological constraints in their area of assignment.

This study aimed to identify organizational issues related to staff nurses’ use of smartphones for work purposes and examine whether such issues influence nurse administrators to support such practice in the Philippines. As a developing country in Southeast Asia, the Philippines is an interesting context for this study because most hospitals in the Philippines do not have sufficient health information technologies that can support health care professionals’ clinical work [28]. Moreover, the country is currently facing a decline in its nursing workforce [29]. Considering that most hospitals in the Philippines lack basic health information technologies and are working under manpower constraints, it is interesting to evaluate the extent to which nurse administrators in the Philippines support smartphone use for mitigating such challenges.

Overall, this study is another contribution based on a series of studies examining the implications of personal smartphone use of nurses in clinical settings in the Philippines [16,18,30]. On a practical note, the findings of this study can help guide health care organizations in developing appropriate BYOD policies for health care staff in the era of IT consumerization.

### Organizational Support Theory

This study draws on the Organizational Support Theory (OST) as its theoretical foundation. OST posits that employees develop beliefs on how organizations support their actions [31]. These beliefs are based on the actions acted upon by organizational agents (ie, top management, immediate superiors, rank and file employees) who exert power over employees. Therefore, employees tend to have perceptions of organizational support, and this can influence their actions, including the use of technology [32,33]. In the context of this study, a recent work in the Philippines that also draws on OST suggests that staff nurses’ perceived organizational support (ie, staff nurses’ perception of organizational support on the use smartphones for work purposes as derived from the hospital management, immediate nursing superiors, fellow staff nurses, and medical doctors) had an indirect effect on their use of smartphones for work purposes [18]. Clearly, this finding shows how crucial organizational support is when IT consumerization occurs in the clinical setting.

In addition to the perceived organizational support, OST also posits that the support exhibited by organizational agents constitutes actual organizational support [31]. According to Rhoades and Eisenberger [34], supervisors are one of the key organizational agents who directly influence employees’ perception of organizational support. This can be attributed to the fact that supervisors have the responsibility to direct and evaluate subordinates [34]. Therefore, the extent of support conveyed by supervisors (ie, actual organizational support) can influence employees’ perceived organizational support. For the purposes of this research, supervisors will be referred to as nurse administrators, who are nurses with supervisory function [35]. In the Philippines, nurse administrators typically include head nurses (entry-level supervisory position; sometimes referred to as charge nurses), nurse managers (midlevel supervisory position), and nurse supervisors (midlevel supervisory position) [16].

Although the link between actual and perceived organizational support is established by meta-analyses that show that supervisor support is a strong positive predictor of perceived organizational support [34,36], the influence of organizational issues on supervisors’ support to employees’ use of technology is currently unclear. The rationale of exploring this aspect in this study is that certain issues might influence nurse administrators’ support to staff nurses’ use of smartphones for work purposes, which then influences staff nurses’ perceived organizational support. Although previous studies have presented several issues related to nurses’ use of smartphones for work purposes (ie, blanket ban of smartphones at work, reduced professionalism, and hospitals not providing smartphones and credits for nurses) [14-16,19-22], it is unknown whether these issues influence nurse administrators’ support to nurses’ use of smartphones for work purposes. For instance, if a hospital implements a blanket ban on smartphones, would a nurse administrator prohibit a staff nurse to use a smartphone if this is the only means possible to contact a physician given that there is no other hospital device to use? As one of the hospital’s organizational agents, nurse administrators need to deal with balancing the risks (eg, privacy and confidentiality concerns) [14,15] and benefits (eg, opportunity to enhance quality of care to patients, faster communication, and information seeking) [16-19] associated with using smartphones for clinical work, especially in low-resource settings. Overall, this study aimed to answer the following research question: *What are the organizational issues that influence nurse administrators’ support to staff nurses’ use of smartphones for work purposes?*

## Methods

### Study Design and Ethics Approval

This study used a qualitative research design because it allows the collection of rich descriptions of organizational issues related to the use of a health information technology [37]. The institutional review board of Nanyang Technological University gave ethical clearance for the study (IRB 2016-09-003). In addition, the administrators or ethics committees of the hospitals where the focus groups were conducted approved the study. All participants provided written and verbal consent to participate in the study. Participants were given Philippine Peso (PHP) 200 (approximately US $4) worth of gift vouchers for their participation.

### Selection and Profile of Focus Group Sites

**Figure 1:**
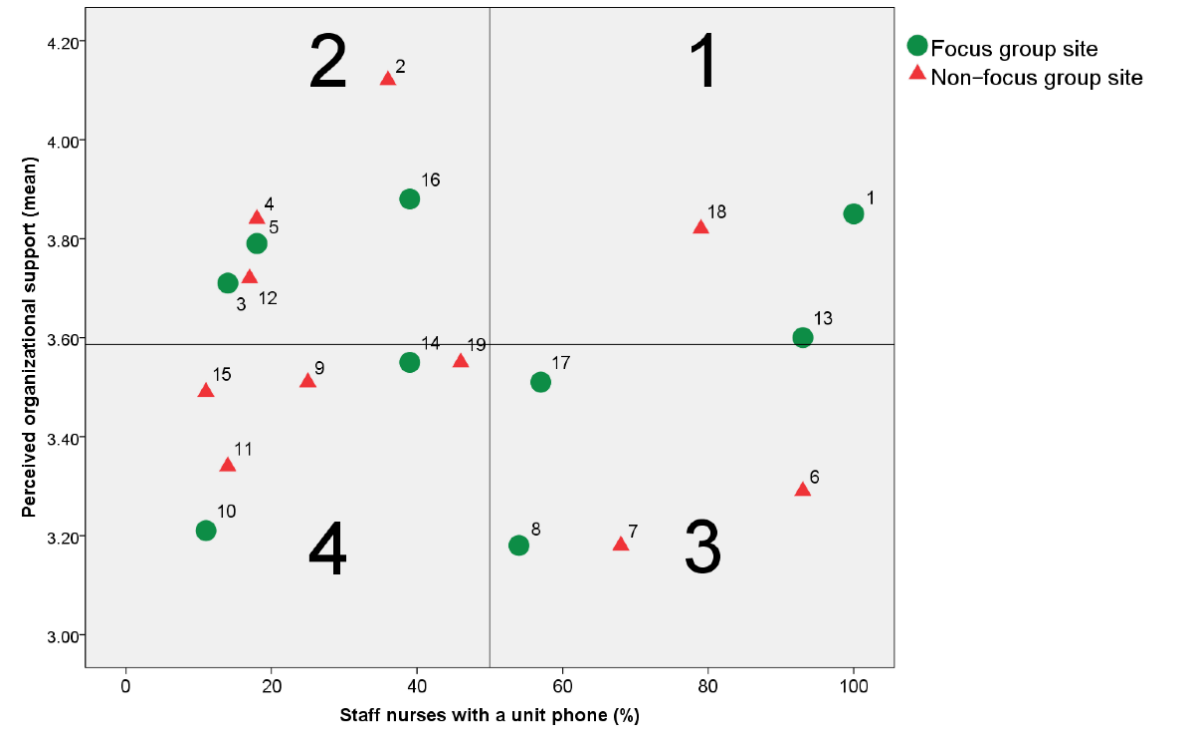
Hospital quadrants and focus group sites (hospital codes are reflected in each dot or triangle; dots represent actual focus group sites).

Overall, 9 of 19 hospitals in Metro Manila, Philippines, that were part of an earlier study [18], were randomly selected using a hospital matrix (Figure 1). The hospital matrix was developed based on data collected for that study (Table 1). To identify hospitals that are within the same quadrant, cutoff values were used as markers in the scatterplot.

Although there is no consensus on the required number of focus group sites [38], at least 2 hospitals per quadrant were selected. To select hospitals, half of the hospitals from each quadrant were randomly selected. Figure 1 shows the hospitals where the focus groups were conducted, and Table 2 provides a summary of the selected hospital’s characteristics. Subsequently, focus groups were conducted in 6 private and 3 government hospitals.

**Table 1:** Hospital matrix data.

ID	Staff nurse respondents, n	Perceived organizational support^a^	Staff nurses with unit phone, n (%)^b^	Focus group site^c^
H1	28	3.85	28 (100)	Yes
H2	26	4.12	9 (35)	No
H3	27	3.71	4 (15)	Yes
H4	27	3.84	5 (19)	No
H5	28	3.79	5 (18)	Yes
H6	27	3.29	25 (93)	No
H7	28	3.18	19 (68)	No
H8	28	3.18	15 (54)	Yes
H9	28	3.51	7 (25)	No
H10	28	3.21	3 (11)	Yes
H11	26	3.34	3 (12)	No
H12	30	3.72	5 (17)	No
H13	27	3.60	25 (93)	Yes
H14	28	3.55	11 (39)	Yes
H15	26	3.49	3 (12)	No
H16	27	3.88	10 (37)	Yes
H17	27	3.51	15 (56)	Yes
H18	27	3.82	21 (78)	No
H19	24	3.55	11 (46)	No

^a^Mean score for perceived organizational support. Maximum score of 5. See items for perceived organizational support in Bautista et al [18].

^b^Each respondent was asked if the hospital provides their work area with a unit phone (ie, hospital-provided mobile phone).

^c^Refers to the hospitals that eventually became focus group sites.

**Table 2:** Characteristics of hospital sites for focus group (N=9).

Quadrant number and code		Location^a^	Ownership	Bed capacity
**1**				
	H1	South	Private	>300
	H13	Central	Private	<300
**2**				
	H3	South	Government	<300
	H5	North	Private	<300
	H16	Central	Government	>300
**3**				
	H8	North	Private	<300
	H17	Central	Private	>300
**4**				
	H10	North	Private	>300
	H14	South	Government	>300

^a^Location within Metro Manila, the Philippines.

### Selection and Profiles of Participants

Consistent with qualitative research design, purposive sampling was used to recruit participants [39]. Eligibility criteria were as follows: (1) aged 21 years or older, (2) have worked for at least a year in their current hospital, and (3) currently working as a nurse administrator. Participants were recruited by coordinating with each hospital’s nursing department. Having nurse administrators from various areas of the hospital ensured maximum variation sampling [40].

Overall, 43 nurse administrators participated in the focus groups (Table 3). They included 22 head nurses, 10 supervisors, 9 nurse managers, and 2 infection control nurses (a supervisory position in the hospitals where they were employed). A total of 9 focus groups were conducted, and each session had 4 to 5 participants. Although focus groups are usually composed of 6 to 12 participants per group [40], it should be small enough for all participants to contribute but large enough to share various opinions [41]. Therefore, having 4 to 5 participants per focus group was enough to obtain rich data [42,43].

In general, participants were mostly female (36/43, 84%), and their median age was 45 years. The median length of service was 16 years, and the median number of nurses supervised was 17. Maximum variation sampling [40] was achieved because the participants represented several general (eg, wards and outpatient department) and specialty (eg, intensive care, operating theater, and emergency department) areas.

**Table 3:** Profiles of focus group participants (N=43).

Quadrant number and code		Hospital ownership	Sex	Age	Work experience (years)	Position	Staff nurses supervised (n)	Area
**1**								
	H1P1	Pvt^a^	F^b^	44	13	HN^c^	17	Intensive care unit
	H1P2	Pvt	M^d^	45	17	HN	13	Medical surgical ward
	H1P3	Pvt	F	46	10	HN	17	Emergency department
	H1P4	Pvt	F	45	14	NS^e^	8	General nursing ward
	H1P5	Pvt	F	48	13	NS	26	Training and research
	H13P1	Pvt	F	57	15	HN	12	Pediatric ward
	H13P2	Pvt	F	52	13	HN	26	Operating/delivery room
	H13P3	Pvt	M	42	8	HN	11	Intensive care unit
	H13P4	Pvt	M	44	10	NS	50	Nursing department
	H13P5	Pvt	F	51	15	HN	10	Neonatal intensive care unit
**2**								
	H3P1	Gov^f^	F	38	17	NM^g^	13	Obstetrics/gynecology ward
	H3P2	Gov	F	49	7	NM	7	Hemodialysis unit
	H3P3	Gov	F	44	24	NM	9	Surgical ward
	H3P4	Gov	F	45	15	NM	13	Pediatric ward
	H3P5	Gov	F	52	20	NM	20	Delivery room
	H5P1	Pvt	F	48	6	NM	100	General nursing units
	H5P2	Pvt	M	32	7	HN	18	Operating theater
	H5P3	Pvt	M	29	5	HN	12	Medical surgical ward
	H5P4	Pvt	M	30	9	HN	20	Intensive care/medical unit
	H5P5	Pvt	F	40	18	NM	100	Special nursing units
	H16P1	Gov	F	34	26	HN	11	Pay ward
	H16P2	Gov	F	35	16	HN	22	Neurology/ear/nose/throat unit
	H16P3	Gov	F	36	41	NS	60	Medicine ward
	H16P4	Gov	F	37	37	NS	29	Emergency department
	H16P5	Gov	F	38	22	HN	16	Outpatient department
**3**								
	H8P1	Pvt	F	29	5	HN	38	General nursing unit
	H8P2	Pvt	F	50	26	HN	9	Neonatal intensive care unit
	H8P3	Pvt	F	45	14	HN	3	Ambulatory care
	H8P4	Pvt	F	39	15	NS	70	Acute and critical care units
	H17P1	Pvt	F	64	41	NS	9	Outpatient department
	H17P2	Pvt	F	43	17	NS	9	Nursing department
	H17P3	Pvt	F	50	25	HN	9	Outpatient department
	H17P4	Pvt	F	39	15	ICN^h^	258	All nursing areas
	H17P5	Pvt	F	28	6	HN	13	Critical care unit
**4**								
	H10P1	Pvt	F	49	28	NM	45	Operating theater
	H10P2	Pvt	F	36	12	HN	30	Newborn services unit
	H10P3	Pvt	F	38	19	HN	16	Pediatric/medical surgical unit
	H10P4	Pvt	F	45	22	HN	24	Delivery room
	H10P5	Pvt	F	53	32	HN	11	Obstetrics/gynecology ward
	H14P1	Gov	M	51	29	NS	100	Operating/delivery/emergency
	H14P2	Gov	F	58	32	ICN	50	All nursing areas
	H14P3	Gov	F	58	34	NS	88	Nursing department
	H14P5	Gov	F	59	35	NM	15	Emergency department

^a^Pvt: private.

^b^F: female.

^c^HN: head nurse.

^d^M: male.

^e^NS: nurse supervisor.

^f^Gov: government.

^g^NM: nurse manager.

^h^ICN: infection control nurse.

### Data Collection Procedure

Focus groups were conducted from June to July 2017 by JB. These were held in a time and location arranged by each hospital’s nursing department. Focus groups were conducted during or after the participants’ shift in their respective hospital’s nursing training office or vacant hospital room. To allow freedom of expression, the rooms were closed, and only the study participants were present inside the rooms during focus groups. An interview guide was used during the focus groups. This interview guide was developed based on the relevant literature on nurses’ use of smartphones at work [16,44-47]. Although there were preset questions, probing questions were also asked for clarification or exploration [48].

The focus groups were conducted using a mix of English and Tagalog languages so that participants could clearly express their sentiments. Aside from verbal responses, nonverbal cues (eg, body language and group dynamics) were recorded in the field notes. To allow greater discussion during focus groups, participants were arranged to sit in a circular pattern [49]. Each focus group lasted about 40 min on average. To uphold privacy and confidentiality, participants were asked not to state their name and workplace during the audio-recorded focus groups. Instead, participants were assigned participant numbers for identifying themselves or others during the focus groups. Moreover, all potentially identifiable information was removed from the transcripts.

### Data Analysis

Audio recordings of each focus group underwent verbatim transcription. The transcription was performed by JB because he moderated the focus groups and could identify each participant’s voice in the recordings. All completed transcripts and field notes were imported in NVivo 11 (QSR International, Melbourne, Australia) for data analysis. To analyze the data, a primary-cycle coding was first conducted to break down the data into smaller pieces [40]. This was performed by conducting an extensive line-by-line open coding in which codes were assigned freely to the data [50]. After primary-cycle coding, a secondary-cycle coding was performed by immersing and reflecting on the existing codes. Subsequently, related codes were categorized into conceptual bins from which organizational issues emerged [40]. Eventually, the issues were grouped based on OST [31] by classifying those that encouraged or inhibited nurse administrators to support staff nurses’ use of smartphones for work purposes. Multimedia Appendix 1 shows the coding table. Discussions among the authors helped identify how themes varied from one case to another in consideration of the participants’ characteristics (eg, hospital type and area). Considering the depth of results, focus group data from 43 participants reached data saturation. To enhance qualitative rigor, steps were taken to enhance its trustworthiness by applying the principles of credibility, transferability, dependability, and confirmability [51]. The following sections present the findings along with relevant quotes.

## Results

### Issues That Encouraged Support

This theme refers to organizational issues that encouraged nurse administrators to support staff nurses’ use of smartphones for work purposes. Textbox 1 provides an overview of this theme.

**Issues that encouraged support.**

Personal smartphones are superior than workplace technologies.

- Landline telephones are unable to contact mobile phones.
- Intercom system is unreliable.
- Incomplete feedback loop with the desktop-based text messaging software.

Personal smartphones resolve unit phone problems

- Absent unit phones.
- Insufficient unit phones.
- Insufficient unit phone credits.

Policy is unrealistic to implement.

- Making exemption.
- Ban on smartphone use only for nonwork purposes.

#### Personal Smartphones Are Superior Than Workplace Technologies

Generally, nurses should use technologies provided by their hospitals for work. Although technologies were limited in most hospitals where the focus groups were conducted, participants shared that their hospital provided a few technologies that they could use to facilitate communication with colleagues. However, such technologies were deemed unreliable and inferior compared with the smartphones that nurses possess. Therefore, nurse administrators were inclined to allow their nurses to use their personal smartphones for work purposes.

#### Landline Telephones Are Unable to Contact Mobile Phones

Every private and government hospital had landline telephones considering that it is one of the most fundamental communication technologies one would have. However, most participants reported that although their hospitals provide landline telephones, staff nurses cannot use them to call mobile phones because these are limited to communication with other landline telephones within the hospitals. As a result, most of the participants allowed nurses to use their smartphones to communicate with members of the health care team:

The options for us to call cellphone, overseas calls, and NDD [national direct dialing] is restricted [in the landline]. It is restricted to all [making out-of-hospital calls].

H5P3, Head Nurse

Hospital 8 was an exception because participants shared that their staff nurses could make mobile phone calls by using hospital landline telephones that connected them to mobile phones through the operator. This is generally useful, but a participant from Hospital 8 noted that when operators were unavailable, nurse administrators would allow their nurses to use smartphones to contact doctors:

My staff has Globe [a service provider] and if we need to inform a Smart [refers to a doctor that is subscribed to another service provider], we will call to the operator. Now, if Smart is not available to the operator, I have one staff [nurse] that has an unli [unlimited call] to Smart. One of my staff is unli to Globe. We just borrow [each other’s smartphone].

H8P3, Head Nurse

#### Intercom System Is Unreliable

Participants from Hospital 3 and Hospital 5 described their intercom system as a local two-way communication system in which a microphone and a loudspeaker were installed in every nursing area. Although this was deployed to help facilitate communication among health care staff within the hospital, some of them noted several problems when using the intercom system that consequently reduced its usefulness. For instance, one participant noted that “it is difficult to use” the intercom system (H3P3, Nurse Manager), and another shared that “sometimes it is busy” (H3P1, Nurse Manager). Conversely, a participant stated that such technology “is actually good” but lamented that it was also an inefficient form of communication technology as it is an indirect means of communication:

With the number of patients being catered by the hospital [many patients] including the number of resident doctors that is rotating in the hospital [few doctors], doctors would not receive our message. So, our requests would take time.

H5P3, Head Nurse

Consequently, the problems experienced by the participants with their intercom system served as a cue for them to allow staff nurses to use their smartphones for work purposes. For instance, one participant stated that they could “directly call the doctors for a referral” using their staff nurses’ smartphone and they “need not bother to press anything on our intercom” (H3P3, Nurse Manager). Similarly, when the intercom system was busy, one participant was relatively fine when her nurses “made calls or texted” (H3P1, Nurse Manager) using their smartphones just to contact their patients’ doctors. Conversely, one participant explained that communication via mobile phones is faster than an intercom system, to the extent that they requested their management to provide them with a unit phone:

For us to mobilize and facilitate information and updates, it is much easier on our phones. It is not because we want to remove the paging [intercom] system but there are times that we need a much faster means of communication. That is why we are requesting a [unit] phone.

H5P3, Head Nurse

#### Incomplete Feedback Loop With the Desktop-Based Text Messaging Software

Participants described the desktop-based text messaging software as a software that allowed nurses to send and receive text messages to and from mobile phones regardless of the service provider. In most situations, staff nurses used them to send patient referrals to doctors. In Hospital 1, participants mentioned that their hospital installed *Maxxtext* in each desktop computer. Similarly, Hospital 17 also has a similar software called *Infotext*.

Despite being an alternative to mobile phones for sending text messages, a major problem with this technology is the difficulty in receiving replies. For instance, one participant shared that her staff nurses in the intensive care unit used *Infotext*, but they were bothered because “the problem with Infotext is we cannot immediately receive the reply” (H17P5, Head Nurse). She also noted that doctors were familiar with this problem, and “it is not guaranteed that they will reply [in the Infotext]” considering that there was a “feedback problem” (H17P5, Head Nurse).

The feedback problem associated with this technology was a strong concern because a patient’s life in the intensive care unit depends on the speed of coordination among the health care team. Considering this problem, one participant shared that instead of using *Infotext*, she allowed her staff nurses to use their smartphones when making referrals to doctors, especially during emergencies:

If there were emergency cases, you cannot avoid not to use your personal phone because residents and consultants send their replies to us. We do not have a cellphone [in the area]. We just use our own cellphone.

H17P5, Head Nurse

Participants in Hospital 1 also noted the feedback problem with *Maxxtext* and why it became unpopular among staff nurses and doctors. According to one participant, *Maxxtext* was quite useful until it had the same feedback problem, and this led to the termination of the software and the deployment of mobile phones in their hospital:

...[T]hey placed Maxxtext, so we did not bother using the cellphones because it was a much better form of communication with the doctors. However, there was a time that Maxxtext had a [feedback] problem, so the [software] contract was not renewed. What they did instead was to give cellphones per unit.

H1P4, Nurse Supervisor

#### Personal Smartphones Resolve Unit Phone Problems

During the focus groups, most participants were highly vocal about unit phone problems, which suggested their importance and relevance. This is understandable as most of the participants came from hospitals where unit phones were mostly not provided to staff nurses. In situations where unit phones were not provided, they were insufficient, or credits were lacking, nurse administrators were inclined to allow their nurses to use their smartphones for work purposes.

#### Absent Unit Phones

Focus groups with the participants revealed that all government hospitals (ie, Hospital 3, Hospital 14, and Hospital 16) did not provide unit phones, and a few private hospitals provided them (eg, Hospital 1 and Hospital 13). Considering that most of the hospitals did not provide mobile phones to their nurses, participants from those hospitals shared that the smartphones of their staff nurses were very useful, and they allowed their use for work purposes. For instance, one participant shared that she allowed her nurses to use their smartphones “to do research on the case of the patient” because she believed that it is “the fastest way for them to look for information regarding the case of the patient that they are handling” (H5P1, Nurse Manager). Similarly, another participant shared that her area was not provided with a unit phone; as a result, she allowed her nurses to use their smartphones considering their usefulness for communication purposes:

From the ambulatory care, I allow the use of their personal mobile phone because a unit phone is not provided by our hospital. They use it to inform doctors if there are admissions.

H8P3, Head Nurse

Apart from the usefulness of smartphones, some participants also shared that smartphones contributed to improving the quality of care rendered to patients. This occurred when smartphones helped nurses to immediately cater to their patients’ needs. As a result, the absence of a unit phone served as a cue for the participants to support their staff nurses’ use of smartphones for work purposes. For example, one participant stated that “the patient benefits from it because they [staff nurses] can facilitate immediate interventions to the patient” (H14P2, Infection Control Nurse). Moreover, one participant shared that although mobile phones can be “a double-edged sword,” he argued that:

From a clinical standpoint, if you will use it in the interest of caring for patients, it will be very beneficial and efficient.

H5P4, Head Nurse

Nurses would need to find ways to provide the best possible service to their patients despite resource constraints. This meant even using their smartphones just to accomplish their task. According to some participants, their staff nurses’ use of smartphones for work purposes is a manifestation of their capability to adapt in a situation where such technology is not provided by their hospitals. For one participant, the absence of a unit phone made him decide to allow its use because it is a way for staff nurses “to gather technical or clinical information outside of our norm or usual routine” (H5P4, Head Nurse). Moreover, another participant explained how nurses adapt to perform their work in the absence of unit phones:

If the organization is unable to provide their needs [like unit phones], it talks about the adaptability of the people working under them. So, of course, if you want to finish your task immediately, you [would] opt to use your own cellular phone.

H5P5, Nurse Manager

Although most participants supported their staff nurses’ use of smartphones for work purposes because of the absence of unit phones in their workplace, a few of them recognized that, in the long term, hospitals should provide unit phones so that nurses would not use their smartphones. This sentiment is best described by a participant from Hospital 5 as he believed that his nurses need a unit phone and having it would result in an outcome where **“**the personal phone [of the nurses] can be kept away and the unit phone is the one outside to be used” (H5P3, Head Nurse). More importantly, another participant expressed that providing her staff nurses with unit phones would be “for the good of the patient” (H16P1, Head Nurse). She added:

It will save us since we can verify and clarify [doctors’ orders] much faster. Patients would not get angry with us that we are not doing anything for them.

H16P1, Head Nurse

Interestingly, the participants also provided details on what mobile phone should hospitals provide to as a unit phone. However, participants were divided on whether a smartphone or a feature phone (nontouchscreen) should be provided. For some participants, providing a feature phone was ideal because it is more durable than a smartphone, and it is not susceptible to theft considering its low value. For instance, one participant shared that her area in the operating room needs “just a keypad cellphone. Like this [points to a feature phone]. It would not get easily destroyed or lost” (H10P1, Nurse Manager). In addition, feature phones could cover most of the staff nurses’ needs because they only frequently used their smartphones to make calls and send text messages to colleagues:

A good situation is for each unit to have one [unit phone]. Just only a keypad phone [refers to feature phone], just for text and call. No camera. No applications. Just a keypad [phone].

H3P1, Nurse Manager

More importantly, feature phones are less likely to be used for nonwork purposes. According to one participant, she preferred a feature phone because this cannot be used to access social media or play mobile games:

Maybe we just need the ones with just keypads [feature phone]. You cannot really avoid that others might use it for FB [Facebook] or games. If it its only keypads, its only for call and text. They are limited to that because it’s the only thing they need in the ward. That should be for all [areas].

H17P3, Head Nurse

On the contrary, some participants indicated a preference to have a smartphone as their unit phone because they intended to use it for documentation purposes. For these participants, being able to take pictures as a means of documentation can reduce their workload and provide visual evidence of certain conditions or events that need to be shown to colleagues as evidence. For instance, one participant shared that “we prefer a touchscreen [smartphone unit phone] because we have referrals that involve pictures and we send them” (H17P5, Head Nurse). Moreover, a participant shared the importance of having a smartphone for documentation in the emergency room:

We need something for documentation because it is important for us. For instance, the patient comes from ER [emergency room]. We endorse the patient in the [other] unit without any bedsore; it needs to be documented, so we need to take a picture of it.

H1P3, Head Nurse

#### Insufficient Unit Phones

Among the focus group sites, only 2 private hospitals (ie, Hospital 1 and Hospital 13) provided most of their staff nurses with a unit phone. These phones were all feature phones that were limited to making voice calls and text messages. However, despite the presence of unit phones in these hospitals, some participants shared that there were occasions on which their staff nurses needed to use their smartphones because not all of them can use the unit phone at the same time. For example, one participant shared that she has more than 17 staff nurses in the telemetry unit, and “nurses could not use the unit phone at the same time. That’s why they use their personal phone” (H1P1, Head Nurse). Likewise, another participant argued that staff nurses’ smartphones are much more accessible to use than the hospital’s unit phones:

We need not share it [refers to their own smartphone]. If the doctor responded, you can easily respond to it without going back to the unit [nurses’ work station] to answer the call from the doctor that you contacted.

H1P2, Head Nurse

Considering that Hospital 13, a private hospital, also had problems with insufficient unit phones in their hospital, one participant suggested that 3 staff nurses can share a unit phone:

It depends on how many are on duty. Sometimes there are three of them because two [staff nurses] plus the charge nurse, so three [nurses per unit phone].

H13P1, Head Nurse

Likewise, when asked how many unit phones might be sufficient for staff nurses in government hospitals, one participant indicated the “three nurses per one unit phone” ratio when she stated that:

Every shift, we are six [staff nurses and nurse administrators]. Maybe have two cellphones.

H16P4, Nurse Supervisor

#### Insufficient Unit Phone Credits

Although Hospital 1 and Hospital 13 provided unit phones as a strategy to reduce staff nurses’ reliance on their own smartphones, participants from these hospitals noted that their hospitals do not necessarily provide them with sufficient credits to use the unit phone. In most cases, unit phones were under prepaid subscription, and credits should be added if consumed completely. Without any credits, unit phones become useless to contact colleagues. As a contingency, participants from Hospital 1 and Hospital 13 allowed their staff nurses to use their own smartphones when their unit phones ran out of credits:

There are times that our load [credits of the unit phone] is already used up, so I allow my nurses to use their own [smartphone].

H13P1, Head Nurse

Similarly, another participant shared that as they are working in the emergency department, they need an immediate response from doctors. Unfortunately, their unit phone “does not always have a load [credits]. It is seldom that it has a load. So, we use our own cellphone” (H1P3, Head Nurse).

Some participants who had unit phones also noted that their hospitals only provided credits through prepaid cards every start of the month, and all of them expressed that this arrangement is not feasible because they can easily consume the credits within a couple of weeks. In most situations, some participants used their own money to purchase credits for the unit phone:

I shoulder the load [credits] for unlicalls [unlimited calls]. I use 95 pesos [About USD 2] per unlicall and text’ that is valid for seven days. After seven days, need to load again. Like that. Really expensive.

H1P3, Head Nurse

An important reason on why credits are consumed quickly is that they often called a member of the health care team (mostly doctors) who had a different service provider. According to one participant, “It is expensive if you are subscribed in Globe to call someone who is subscribed to Smart” (H1P2, Head Nurse). In the Philippines, service providers charge more when users make voice calls to other service providers. For example, Globe Telecom [52] and Smart Communications [53] charge PHP 6.50 (about US $0.13) per minute for calling those with the same service provider and PHP 7.50 per minute (about US $0.15) to others. To avoid potential costs, some participants would ask who among their staff nurses has a smartphone with the same service provider as the one used by the intended recipient of the message or voice call. For example, one participant shared that they “use the cellphone provided by the management but sometimes we use our own cellphone because the line [service provider] is different” (H13P2, Head Nurse).

#### Policy Is Unrealistic to Implement

All 9 hospitals had policies on the use of mobile devices, which were written in hospital memos. Accordingly, hospitals can be divided based on the level of restriction placed on mobile devices. The first group is composed of 4 hospitals (ie, Hospital 5, Hospital 10, Hospital 13, and Hospital 17) that implemented a ban on the use of any mobile devices (whether for work or nonwork purposes) during working hours. It is interesting to note that all these hospitals were private institutions, and only Hospital 13 provided most of their nurses with unit phones. Conversely, the second group comprised 5 hospitals (ie, Hospital 1, Hospital 3, Hospital 8, Hospital 14, and Hospital 16) where the use of smartphones is banned for nonwork purposes but is allowed for work purposes.

#### Making an Exemption

Although Hospital 5, Hospital 10, Hospital 13, and Hospital 17 placed a ban on the use of any mobile devices, participants from these hospitals stated that they make an exemption by allowing staff nurses to use their smartphones for work purposes. This could be attributed to the fact that such policy was unrealistic considering that their hospitals did not provide staff nurses with relevant work-related technologies, such as mobile phones. According to one participant, although their hospital banned the use of any mobile devices, she shared that “you cannot avoid not to use it [smartphones] because it is a big help for nurses in terms of communication, especially when the doctors are not here” (H13P1, Head Nurse). Similarly, another participant shared that “we allow [the use of smartphones] if [it is] related to work, but it is not allowed if you would just use Instagram” (H17P5, Head Nurse).

For most participants, a blanket ban on smartphone use is difficult to implement as these devices were useful and necessary at work. One participant argued that “it is not absolute that we cannot use our phone” considering that “there is a need for us to use the phone [for work purposes]” (H5P3, Head Nurse). Moreover, another participant shared that a blanket ban on smartphones “is not realistic even there is a memo because it is difficult to enforce it” (H17P2, Nurse Supervisor). In Hospital 10, although they strictly implemented a ban on using mobile devices at work (this hospital has one of the lowest mean scores for perceived organizational support), a participant from that hospital argued that:

You cannot avoid not to use [smartphones for work purposes] especially during emergency cases. The ban for us is mostly for personal use.

H10P3, Head Nurse

Overall, there was a consensus among participants that the only time that they can implement a blanket ban on the use of mobile devices is when hospitals can provide sufficient technologies for staff nurses. An indication for this is when staff nurses need not use their smartphones completely for work purposes:

We need more [unit] phones so that they [nurses] can avoid using their personal phone. That is the time that they can fully implement a policy about no use of personal phone in the unit during duty hours.

H1P1, Head Nurse

#### Ban on Smartphone Use Only for Nonwork Purposes

On the contrary, 5 hospitals (ie, Hospitals 1, Hospital 3, Hospital 8, Hospital 14, and Hospital 16) had memos where the use of smartphones is banned for nonwork purposes but is allowed for work purposes. Of these hospitals, 2 are private (Hospital 1 and Hospital 8) and 3 are government hospitals (Hospital 3, Hospital 14, and Hospital 16).

Interestingly, although private hospitals such as Hospital 1 and Hospital 8 provided most of their nurses with unit phones, participants noted that their hospital still allowed staff nurses to use their smartphones for work purposes. Even though the participants described that their memos do not provide a definite list on how it should be used for work purposes, nurses could use their smartphones when there is an urgent need to communicate with colleagues (eg, sending text messages or making calls to colleagues). For example, one participant noted that their hospital issued a *Doctor’s Notification Protocol* (H8P1, Head Nurse) as a basis for them to use their smartphones for work purposes. A colleague of that participant clarified that this protocol allowed nurses to use their smartphones to “inform doctors thru text [messages]” as the hospital had revised the old protocol “by including SMS messaging” as part of the protocol (H8P3, Head Nurse).

In Hospital 1, although unit phones were provided, a participant expressed that their staff nurses can still use their smartphones “if there are important calls or emergencies” related to work (H1P4, Nurse Supervisor). Furthermore, another participant from Hospital 1 shared a policy that allows them to use their smartphones aside from unit phones in the emergency room:

The hospital requires that our referral needs to be answered [by the doctors] within 15 minutes. So, it is important to for us to call [using their own smartphone]. Texting is not reliable because sometimes it [the referral] is received late.

H1P3, Head Nurse

Considering that government hospitals lack adequate technologies for nurses to use, participants from Hospital 3, Hospital 14, and Hospital 16 noted that their hospital allowed the use of smartphones for work purposes and only prohibits their use for nonwork purposes. Although some participants noted that this is not an ideal policy and is the result of their hospitals’ lack of health information technologies, they noted that it is a policy that enables health care staff to properly perform their duty despite resource constraints:

Actually, we have a memo from our chief nursing officer that using cellphone is prohibited particularly for personal use. But, definitely, our nurses can use the cellphone in referring our patients particularly in emergency cases. Now, let us say we caught them using their cellphone, we know that they are not using it for their personal consumption but definitely for referring our patients.

H14P1, Nurse Supervisor

### Issues That Inhibited Support

This theme refers to organizational issues that inhibited nurse administrators from supporting staff nurses’ use of smartphones for work purposes. Textbox 2 provides an overview of this theme.

**Issues that inhibited support.**

Smartphone use for nonwork purposes

- Feelings of frustration and unprofessionalism
- Negative outcomes
- Disciplinary actions

Misinterpretation by patients

#### Smartphone Use for Nonwork Purposes

Although the participants allowed staff nurses to use their smartphones for work purposes, they were equally concerned that some were abusing such considerations by secretly using their smartphones for nonwork purposes. This eventually led them to inhibit support to staff nurses’ use of smartphones for work purposes.

#### Feelings of Frustration and Unprofessionalism

When participants discussed details on staff nurses’ use of smartphones for nonwork purposes, most of them showed a facial expression akin to frustration. This is expected because participants mostly shared statements that reflected frustration when discussing this issue. For instance, one participant shared her frustration over the non–work-related use of smartphones by staff nurses:

If you allow them to use cellphone [for work purposes], some are abusive. Sometimes they will tell you that they are trying to contact the doctor, however, what they are really doing is using it for FB [Facebook], playing games, [or] Instagram.

H17P2, Nurse Supervisor

Apart from feelings of frustration, some participants felt that such behavior did not look like what a staff nurse ought to be doing during the performance of his or her duty. As mentioned by one of the participants, although it is fine for staff nurses to use smartphones for work purposes, its use for nonwork purposes “does not look professional” (H14P1, Nurse Supervisor).

#### Negative Outcomes

Staff nurses’ use of smartphones for nonwork purposes can lead to negative outcomes. Some participants shared that there were times that they did not want their nurses to use their smartphones because they become distracted. This sentiment is described by one participant:

There are times that I do not want them to use their phone because, in just a moment, they have time to chat and [play mobile] games.

H13P5, Head Nurse

Another outcome that participants were concerned regarding the use of smartphones for nonwork purposes is reduced work productivity. This concern was reasonable because all of them believed that smartphone use for nonwork purposes is highly distracting and can result in productivity loss:

When you see [them], you will think that they are just looking for something [that is related to work] but they are just playing games. It is not very good when it comes to work. Of course, if we are in the ward, we need to work. That is one bad impact of it. That is true because it slows down their work.

H3P2, Nurse Manager

For some participants, its use for nonwork purposes also reduced the quality of care because smartphones take away the attention that should have been given to patients. One culprit for this is the use of social media during working hours:

Today, they are not sleeping anymore, but they are using social media, [like] Facebook, during graveyard shift [10pm-6am]. Later, you do not realize that you enjoy browsing and that you forgot that the patient has a due [order]. The work gets delayed, other routines for the patient get delayed. So, the quality of care is affected.

H1P1, Head Nurse

#### Disciplinary Actions

Participants noted that they enforced disciplinary actions when they caught staff nurses using their smartphones for nonwork purposes. For most of the participants, the first thing that they did was to give verbal reminders that included some counseling. For instance, one participant shared that “if I caught them playing games, I remind them that we have a memo that using cellphone is not allowed in the operating theatre” (H10P1, Nurse Manager)*.* Similarly, another participant mentioned that she usually calls the attention of her nurses and reminds them that they “have a policy that cellphones are not allowed during their tour of duty” (H17P2, Nurse Supervisor).

Consequently, some participants shared that they implemented preventive measures, such as asking nurses to place their smartphones inside lockers or cabinets. By asking the nurses to put it inside lockers prevented them from placing it in their pockets, which then reduced the tendency for it to be used for nonwork purposes. For instance, one participant mentioned that “their phone should not be even in their pockets. It should be in the locker” (H1P1, Head Nurse). In situations that there is a need for nurses to use their smartphones, they can get it from their locker:

We do not allow their own cellphone inside the OR [operating theatre]. They need to place it inside the locker. But sometimes, their phone rings and they need to pick it up, then they go to pick it up.

H13P2, Head Nurse

To some extent, some participants shared that their hospital ordered nurse administrators to implement harsh disciplinary actions. Accordingly, if verbal reminders were not enough for repeat violators, nurse administrators could confiscate the smartphone as the next step:

I usually call their attention [upon seeing nurses using smartphones for non-work purposes]. Then I ask them to work on our stocks [materials in the operating theatre]. To some extent, for repeat violators, we confiscate their cellphones and we give it back after duty.

H5P5, Nurse Manager

Aside from confiscation, participants in Hospital 10 asked violators to pay a fine when caught using smartphones for nonwork purposes. As mentioned by a participant from Hospital 10, “We have a fine of 100 pesos [about USD 2] then we confiscate the cellphone. They can get that after duty” (H10P1, Nurse Manager). Nonetheless, the hospital also made a record of such violations by asking staff nurses to file an incident report. For instance, another participant from Hospital 10 shared that “in our area, there is [a need to file] an incident report” (H10P2, Head Nurse). These findings somewhat indicate why Hospital 10 had one of the lowest perceived organizational support scores among all hospitals. Finally, there were instances that staff nurses were suspended from work as they were caught using smartphones for nonwork purposes:

There was an instance where the chief nurse caught some of our nursing staff watching something on their mobile phone and disciplinary action was given. It was work suspension. Three days for each [nurse].

H5P4, Head Nurse

#### Misinterpretation by Patients

Most of the participants shared that they cautioned staff nurses when using smartphones in front of patients as there is a tendency for patients to interpret that staff nurses use their smartphones for nonwork purposes. This issue was expressed mostly by participants from private hospitals because they cater to *pay patients*. Accordingly, pay patients tend to expect a higher standard of service than patients admitted in government hospitals where most are subsidized. This means that patients in private hospitals are relatively observant on how staff nurses conduct their work. For instance, a participant from Hospital 17 (a private hospital) shared that:

It is normal in my ward that a patient becomes angry because they thought that our staff [nurses] are texting [for personal use]. However, in that case, the nurse was only using it to count the drops of the IV [intravenous] fluids.

H17P2, Nurse Supervisor

On the contrary, patients who are sick or in pain are generally sensitive, and they may easily complain when they feel neglected, especially when staff nurses use their smartphones, regardless of whether it is for work or nonwork purposes. For example, one participant shared that her patients in the delivery room “are in labor...in pain, so they are really sensitive,” as a result, “if they see that you are holding your cellphone and you did not immediately address their need, these result in complaints” (H5P5, Nurse Manager).

Although it is difficult for the participants to oblige their staff nurses not to use their smartphones considering how necessary it is in their work, they advised them to use it discreetly and outside the view of patients. The aim of this advice is to ensure that patients do not feel neglected when staff nurses use their smartphones even it is for work purposes. For instance, one participant advised her staff nurses to use their smartphones “not in front of the patient” and should there be a need to use it, “they should hide so that the patient would not see them” (H14P3, Nurse Supervisor). In addition, another participant shared a vivid explanation of some considerations when staff nurses use their smartphones for work purposes:

What I advise them is to use it discretely and not obvious especially when there are other people walking and you look like doing nothing but just using the cellphone. If there are [work-related] calls, I would ask them to hide either in the CR [comfort room] or in our dressing room. Sometimes, nurses are doing work then the phone rings. They are not allowed to answer it since they are in front of other people. So, they need to hide.

H10P5, Head Nurse

## Discussion

### Issues That Encouraged Nurse Administrators to Support Nurses’ Use of Smartphones for Work Purposes

One of the key findings is that a hospital’s lack of adequate health information technologies can drive (or force) nurse administrators and staff nurses to be resourceful in using an existing technology that they can use in their work regardless of policy constraints. This is somewhat expected in most, if not all, hospitals in the Philippines because the deployment and implementation of even the most basic forms of health information technologies (eg, electronic health records) are lagging [28].

In the context of this study, problems encountered by nurse administrators with existing hospital communication technologies (eg, landline telephones and intercom) served as a justification for them to allow their nurses to use smartphones for work purposes. As a result, this led nurse administrators to support the use of smartphones for work purposes to overcome problems associated with existing workplace technologies. This is expected considering that nurses have a moral responsibility to take care of patients, and technologies, such as smartphones, can serve as a bridge to address health care gaps, especially in low-resource settings [21,54].

Apart from problems with existing workplace technologies that made personal smartphones more superior, the absence of unit phones, and its credits, served as another reason for nurse administrators to allow staff nurses to use smartphones for work purposes. Similarly, when unit phones lack the necessary credits to be functional, nurse administrators have no choice but to allow their staff nurses to use their smartphones. Although the issue regarding the absence of unit phones is expected in most hospitals in developing countries [54], it is interesting to note that studies conducted in locations where health information technologies are expectedly robust, such as in Australia [55] and Taiwan [19], also showed that nurses used their smartphones for work purposes because it is not provided to them by the hospital. Overall, the findings indicate that smartphones are now essential in the work of staff nurses, and nurse administrators would allow their use, especially when hospitals do not provide adequate unit phones and credits to their nurses.

Another key finding of this study is that a blanket ban policy on mobile devices did not deter nurse administrators’ decision to allow staff nurses to use smartphones for work purposes because such policy was perceived to be unrealistic. In general, the findings are contrary to previous studies in which nurse administrators tend to be unsupportive of nurses’ use of smartphones [14,15], considering that nurse administrators in this study were generally supportive of smartphone use as long as it was used solely for work purposes. Although allowing staff nurses to use smartphones for work purposes contradicts a hospital’s blanket ban policy on mobile devices, this policy can only be implemented realistically if a hospital provides nurses with adequate technologies. Unfortunately, this is not the case for most hospitals in the Philippines considering that deployment of health information technologies is relatively low [28].

Recognizing the limitations present in their workplace, nurse administrators tend to take a pragmatic approach to BYOD policies by implementing the ban only for smartphone use for nonwork purposes. Therefore, hospitals that implement a blanket ban on mobile devices and do not provide adequate technologies to their nurses will have a difficulty in implementing such a policy, and it is expected that there will be a disconnect between policy and practice [14]. As argued by Johansson et al [56], the use of smartphones by nurses is a means to support their practice and is not primarily an outcome of policies implemented by hospitals. Although the findings are generally reflective of circumstances in developing countries [8,21], the disconnect between policy and practice regarding smartphone use among nurses is also a concern in developed countries, such as in the United States [14,57], the United Kingdom [46], Canada [58], Australia [55], and Italy [22]. As argued by Flynn et al [57], the disconnect between policy and practice implies that hospital administrators should develop and implement realistic policies that recognize the increasing role of smartphones in clinical practice.

### Issues That Inhibited Nurse Administrators to Support Staff Nurses’ Use of Smartphones for Work Purposes

Although staff nurses generally used their smartphones for work purposes, it is inevitable that some would use it for nonwork purposes, such as playing mobile games, making personal calls and text messages, and accessing social media [14-16,18,20,22,44,58]. This tends to result in feelings of betrayal and perceptions of unprofessional behavior. According to researchers [14,15], the prospect of nurses using their smartphones for nonwork purposes is unprofessional because it does not align with the ethical and legal standards that define the profession.

Nonetheless, for nurse administrators, the use of smartphones for nonwork purposes is a concern because it is a prime source of distraction that can reduce productivity and the quality of care rendered to patients [20-22,44,45]. This finding is consistent with a recent study in which non–work-related use of smartphones was found to be negatively associated with perceived quality of care and perceived work productivity [18]. Given these negative outcomes, it is expected that some nurse administrators may not support nurses’ use of smartphones, whether it is for work or nonwork purposes.

The negative outcomes resulting from nurses’ use of smartphones for nonwork purposes also led nurse administrators to enforce disciplinary actions against offenders. Similar to the findings of the study by Brandt et al [14], most nurse administrators tend to use verbal reminders and counseling as first-level interventions. Other disciplinary actions were also implemented, such as placing smartphones inside the locker, confiscation, and, to a certain extent, suspension [14]. However, what is novel in this study was the implementation of fines as no previous study has documented this form of disciplinary action. Although a fine of PH*P* 100 (about US $2) might seem little, this is a significant amount of money for staff nurses in the Philippines because it can constitute about one-fifth of their daily wage. In general, the results show that nurse administrators do not tolerate the use of smartphones for nonwork purposes, and they implement various forms of disciplinary actions to deter non–work-related use of smartphones.

Another issue found in this study was that nurse administrators were conscious of patients misinterpreting nurses’ use of smartphones. Such a concern was reflected in studies in the United States [59], Canada [58], and Sweden [56]. Although this issue did not prompt nurse administrators to completely ban the use of smartphones among their nurses, they advised the nurses not to use it in front of patients to reduce the chances of receiving complaints. A potential reason for giving such advice is that nurse administrators must maintain a good nurse-patient relationship. According to Pullen and Mathias [60], an essential aspect of this relationship is the preservation of mutual respect between the nurse and the patient. Considering that nursing is a patient-facing work [59], it is important for nurse administrators to make sure that nurses give patients the highest possible level of respect.

### Limitations

This study recognizes that the organizational issues were limited to focus groups with nurse administrators. Ideally, these issues should come from interviews with a variety of hospital stakeholders (eg, health care professionals, hospital administrators, and patients). As a recommendation, future studies can be geared toward including other health care professionals when it comes to identifying organizational issues related to nurses’ use of smartphones for work purposes.

### Conclusions

This is one of the few studies to use OST as a framework to examine the influence of organizational issues on organizational support within the context of IT consumerization in clinical settings. Therefore, future studies can incorporate this theory when examining organizational issues brought upon by IT consumerization. Apart from theoretical insights, the study can be used as a basis for developing appropriate BYOD policies in organizations where IT consumerization is an organizational issue. Although the findings are in the context of health care, these can also be applicable to non–health care organizations where IT consumerization is prevalent.

## Appendix

Multimedia Appendix 1 Coding table.

## Abbreviations

BYOD: bring your own device
OST: Organizational Support Theory
PHP: Philippine Peso
