# Supplementary material for: Influence of Organizational Issues on Nurse Administrators’ Support to Staff Nurses’ Use of Smartphones for Work Purposes in the Philippines: Focus Group Study
Source: JMIR Nurs. 2020 Jan 10;3(1):e17040. doi: 10.2196/17040 (PMC8279451; doi:10.2196/17040)
Supplement: Multimedia Appendix 1 [file nursing_v3i1e17040_app1.docx]

Multimedia Appendix 1. Coding Table

Theme 1 – Issues that encouraged support

| First level codes | Second level codes | Categories |
| --- | --- | --- |
| Landline telephones only works for landline telephones within hospital | Landline telephones are unable to contact mobile phones | Personal smartphones are superior than workplace technologies |
| Landline telephones can contact mobile phones but operator is busy or unavailable |  |  |
| Intercom system is difficult to use | Intercom system is unreliable |  |
| Intercom system is an indirect means of communication |  |  |
| Desktop-based text messaging software cannot receive reply | Incomplete feedback loop with the desktop-based text messaging software |  |
| Nurses and doctors do not use the desktop-based text messaging software |  |  |
| Unit phone not provided by hospitals | Absent unit phones | Personal smartphones resolve unit phone problems |
| Personal smartphones are useful |  |  |
| Need to adapt |  |  |
| The need to provide unit phones |  |  |
| Unit phone provided but insufficient | Insufficient unit phones |  |
| Sharing of unit phones |  |  |
| The need to provide enough unit phones |  |  |
| Insufficient credits | Insufficient unit phone credits |  |
| No credits |  |  |
| Telecom charges in the Philippines |  |  |
| Paying out of the pocket for credits |  |  |
| Personal smartphones are beneficial at work | Making exemption | Policy is unrealistic to implement |
| Blanket ban is difficult to implement |  |  |
| Blanket ban is implementable when sufficient technology is present |  |  |
| Policy adjustments | Ban on smartphone use only for non-work purposes |  |
| Allowed for work-related emergency calls and text messaging |  |  |
| Not an ideal policy but practical |  |  |

Theme 2 – Issues that inhibited support

| Frustration when smartphones are allowed to be used for work purposes but is used for non-work purposes | Feelings of frustration and unprofessionalism | Smartphone use for non-work purposes |
| --- | --- | --- |
| Use of smartphones for non-work purposes is unprofessional |  |  |
| Distraction | Negative outcomes |  |
| Reduced work productivity |  |  |
| Reduced quality of care |  |  |
| Preventive measures | Disciplinary actions |  |
| Verbal reprimand |  |  |
| Confiscation |  |  |
| Paying fines |  |  |
| Work suspension |  |  |
| Patients who misinterpret nurses’ use of smartphones |  | Misinterpretation by patients |
| Situations when patients misinterpret nurses’ use of smartphones |  |  |
| Strategies to prevent misinterpretation |  |  |
